# A 25-Residue Peptide From *Botrytis cinerea* Xylanase BcXyn11A Elicits Plant Defenses

**DOI:** 10.3389/fpls.2019.00474

**Published:** 2019-04-16

**Authors:** Marcos Frías, Mario González, Celedonio González, Nélida Brito

**Affiliations:** Área de Bioquímica y Biología Molecular, Departamento de Bioquímica, Microbiología, Biología Celular y Genética, Universidad de La Laguna, San Cristóbal de La Laguna, Spain

**Keywords:** xylanase, elicitor, PAMP, plant defense, hypersensitive response (HR)

## Abstract

Plants activate defense responses against a possible pathogen once pattern-recognition receptors (PRRs) perceive the presence of pathogen-associated molecular patterns (PAMPs). Glycosyl hydrolase family 11 (GH11) endoxylanases from *Trichoderma, Fusarium* and *Botrytis* species have been described as being able to induce the defense response in plants, in a way that is independent of its enzymatic activity. However, until now, it has not been possible to establish with certainty which regions of these enzymes are recognized by plants as PAMPs. We show here for the first time that a short 25-residue peptide (named Xyn25) from the *Botrytis cinerea* xylanase BcXyn11A can reproduce by itself all the effects observed for the treatment of plants with the whole BcXyn11A protein. These include necrosis on leaves, seedling growth inhibition, induction of a ROS burst, electrolyte leakage, cytoplasm shrinkage, autofluorescence, cell death, and induction of defense genes. Two highly conserved four-amino acid regions within Xyn25 were shown to be necessary for the elicitation activity by substituting them with tracts of four alanine residues.

## Introduction

The β-1, 4-endoxylanase BcXyn11A is secreted by the phytopathogenic fungus *Botrytis cinerea* (Brito et al., 2006). This necrotrophic ascomycete is considered the second most important plant pathogen according to its economic and scientific importance (Dean et al., 2012) and its infection strategy includes the induction and modulation of the Hypersensitive Response (HR) in the host. This process culminates with the necrosis of plant tissues in the area surrounding the infection, thus generating dead tissue for this necrotroph to grow on (Govrin and Levine, 2000; Mengiste, 2012). BcXyn11A is one of the factors involved in the induction of HR in plants, and thus in *B. cinerea* virulence (Noda et al., 2010). This enzyme hydrolyses the linear backbone of xylan (Brito et al., 2006; Noda et al., 2010), the main hemicellulose component of the plant cell wall (Pollet et al., 2010), and it is expressed early during the *B. cinerea*-tomato plant interaction and remains stable even at 3 days after inoculation (Brito et al., 2006). The protein is also able to induce necrosis of the plant tissues, as well as the production of reactive oxygen species (ROS), when infiltrated in tomato and tobacco leaves (Noda et al., 2010). Four different versions of BcXyn11A lacking enzymatic activity were obtained by site directed mutagenesis (E112Q, E122S, E214Q, and E214S) and all of them triggered a response in plants similar to that of BcXyn11A, indicating that the eliciting activity of the protein is not related with its ability to hydrolyze xylan (Noda et al., 2010). On the other hand, BcXyn11A has also been shown to contribute to the virulence of the fungus, as Δ*Bcxyn11A* mutants are less pathogenic than the wild type (Brito et al., 2006) and the retransformation of these mutants with the *Bcxyn11A* gene restored the wild-type virulence (Noda et al., 2010). Any of the four new versions of the protein lacking xylanase activity was able to complement the Δ*Bcxyn11A* mutant phenotype (Noda et al., 2010), indicating that the contribution of BcXyn11A to the virulence of *Botrytis* is due to its necrotizing activity and not to its endoxylanase activity.

The necrotizing activity of xylanases from glycosyl hydrolase family 11 (GH11) has been well characterized for EIX (Ethylene-Inducing Xylanase) from *Trichoderma viride* (Dean et al., 1989). EIX is known to be recognized as a PAMP by two plant pattern receptors in tomato cells, LeEix1, and LeEix2, both considered as leucine-rich-repeat receptor-like-proteins (LRR-RLP), although only the latter is able to generate a defense response upon EIX binding, triggering the HR, ethylene production, and other plant defense mechanisms (Ron and Avni, 2004). LeEix1 acts as a decoy receptor and has been proposed to be involved in signal attenuation after a long exposure of tomato cells to xylanase (Bar et al., 2010, 2011). Along with EIX and BcXyn11A, other fungal GH11 xylanases have been shown to induce a defense response in plants including *T. reesei* xylanase II (Enkerli et al., 1999) and *F. gramineanum* GH11 xylanases FG_03624 and FGSG_10999 (Tundo et al., 2015).

As far as we know, two reports have proposed short xylanase peptides in the protein surface of EIX and BcXyn11A, away from the active site, to be the regions recognized by the plant pattern receptors. Rotblat et al. (2002), using a combinatorial phage display screening, identified the sequence TKLGE as a region of EIX essential for the elicitor activity. Indeed, when the peptide TKLGE was substituted by VKGT, the protein lost the elicitation activity but not the enzymatic activity. Regarding BcXyn11A, a 30-aa fragment displayed on the enzyme surface, which includes the pentapeptide described in EIX as well as six amino acids perfectly conserved in the three xylanases described as elicitors at that moment (Noda et al., 2010), was able to cause necrosis in tomato leaves and to bind to the membrane of tobacco protoplasts. This effect could only be seen when this 30-aa region was fused to GFP. However, in both reports the treatment of plant cells with the proposed elicitor peptides did not trigger any plant response. Here we report, for the first time, a short 25-amino acid peptide from the xylanase BcXyn11A that is able to elicit a defense response in plants by itself. In addition, we found two conserved regions of four amino acids within this peptide that are required for triggering the complete activation of the host response.

## Materials and Methods

### Homology Modeling of BcXyn11A Structure

A BLAST search against the RCSB Protein Data Bank^1^ using the mature *B. cinerea* BcXyn11A sequence as query, revealed the *Chaetomium thermophilum* xylanase Xyn11A (Hakulinen et al., 2003) as the most similar protein sequence with known structure (67% identity, 81% similarity). This structure (PDB ID-code 1H1A) was then used as template for the homology modeling of BcXyn11A at the SWISS-MODEL website^2^, using the default parameters. The resulting protein structure was then visualized with PyMOL Molecular Graphics System (Version 1.8, Schrödinger, LLC).

### Expression and Purification of BcXyn11A and Xyn60 in *Pichia pastoris*

**FIGURE 1:**
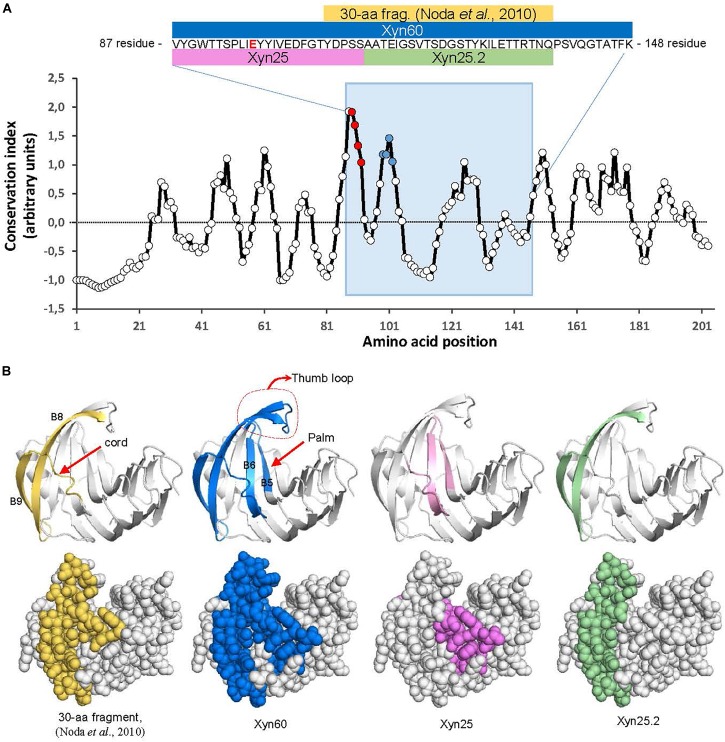
Location of BcXyn11A peptides in the protein sequence and 3D model. **(A)** Evolutionary conservation of the whole BcXyn11A mature sequence calculated with AL2CO (http://prodata.swmed.edu/al2co) for an alignment of 1469 GH11 xylanases downloaded from Pfam (https://pfam.xfam.org) and aligned with Clustal Omega (https://www.ebi.ac.uk/services). Conservation indexes were averaged in windows of five residues to smooth the graph. The position of the Xyn60 peptide is indicated with a blue background. Inset shows the corresponding sequence to Xyn60 (residues 88 to 147 of BcXyn11A sequence expressed in *P. pastoris*, Supplementary Figure S1) and the relative position along the BcXyn11A sequence of the peptides used in this study. One of the two Glu residues (E98) involved in catalysis (Noda et al., 2010) is shown in red. The position of the M1and M2 regions (see below) are pointed as red and blue points, respectively. **(B)** Location of the peptides in a 3D model of BcXyn11A obtained *in silico* (Biasini et al., 2014). Notable structural elements, the thumb, the cord and the palm (Paes et al., 2012) are indicated.

For protein expression, the Easy Select *Pichia* Expression Kit (Invitrogen, United States) was used following manufacturer’s instructions. *Phusion* High-Fidelity DNA Polymerase (New England Biolabs, United States) was used for amplification of DNA sequences to be cloned, and *Taq* polymerase (GenScript, China) was selected for diagnostic PCRs. Oligonucleotides (Supplementary Table S1) were from Life Technologies (United States). Briefly, A 609-bp DNA fragment containing the whole ORF of *Bcxyn11A* gene (acc. no. Bcin03g00480 at^3^), without its own signal sequence (identified with the SignalP 4.1 server at^4^), as well as a 180-bp fragment encoding the central 60-amino acids of the protein (Xyn60, Figure 1), were amplified from *B. cinerea* B05.10 cDNA using the primer pairs Xyn11A-EcoRI-FW/Xyn11A-XbaI-RV and Xyn60aa-EcoRI-FW/Xyn60aa-XbaI-RV, respectively. The obtained PCR products were cloned into pPICZα-A vector, at the *Eco*RI and *Xba*I restriction sites, to generate plasmids pXyn11A and pXyn60, respectively. Competent cells of *Pichia pastoris* KM71H were then transformed with 5 μg of each vector, previously linearized with *Sac*I. Zeocin-resistant transformants were checked by PCR for the right integration of the transforming DNA with the primer pair AOX 5′/Xyn11A-XbaI-RV, in the case of the whole BcXyn11A, or AOX 5′/Xyn60aa-XbaI-RV, in the case of Xyn60. Selected transformants were then sequenced using the AOX 5′primer, and one transformant with the expected sequence from each transformation was selected for expression and purification of the respective protein/peptide. The induction of the expression and the purification of the two polypeptides were carried out as explained before (Frías et al., 2011). The resulting peptide/protein consisted of a fusion displaying c-*myc* and 6xHis motifs at the C-terminus, and their theoretical molecular mass (used to calculate molar concentrations) were 24.33 kDa for recombinant BcXyn11A and 9.2 kDa for Xyn60. About 3–5 mg of each protein were obtained from a total of 200 mL of culture supernatant. The purification of the proteins was verified by Coomassie blue staining in SDS PAGE (Supplementary Figure S1). The Xyn25, Xyn25.2, Xyn25.M1, and Xyn25.M2 peptides were synthetized chemically by GenScript. All protein/peptides used in this work were dissolved in water at the indicated concentrations. Protein concentrations were determined by Bradford (1976), using BSA as standard.

### Quantitative Real-Time PCR (qRT-PCR)

The expression of four different tobacco defense-related genes (*HSR203J*, *HIN1*, *PR1a*, and *PR5*) was analyzed by qRT-PCR. At the indicated times, total RNA was isolated from peptide/protein- infiltrated areas of tobacco leaves, using the RNeasy plant mini kit (Qiagen). Possible contamination with genomic DNA was removed by treatment with RNase-free DNaseI (Genescript) and cDNA was synthetized with the iScript cDNA Synthesis Kit (BioRad) using 1 μg of the purified RNA as template. Amplifications were carried out with the Bio-Rad iQ SYBR Green supermix and the primers listed in Supplementary Table S1, in a CFX96 Real-Time PCR system (Bio-Rad). The tobacco actin gene *Tac*9 was used to verify that equal amounts of RNA were used. Relative expression was calculated for each gene by the ΔΔCt method, and standard deviation (SD) for three independent Ct determinations was calculated using the expression 2^−(Ct±SD)^ (Schmittgen and Livak, 2008).

### Characterization of Plant Defense Symptoms

Leaves from 4 to 5 weeks old *Nicotiana tabacum* (cv. Havana) and *Solanum lycopersicum* (cv. Moneymaker) plants were infiltrated with BcXyn11A or derived peptides at the indicated concentrations, using 1-ml syringes without needle. Ion leakage, cell death (by trypan blue uptake), and cell autofluorescence were determined as previously described (Frías et al., 2011). ROS were determined according to Roux et al. (2011). Briefly, disks of 3.8-mm in diameter were cut from intact tobacco or tomato leaves, transferred to 96-well microtiter plates and immersed in water overnight. Water was then replaced by a solution containing 17 μM BcXyn11A or derived peptides, 17 mg/ml luminol and 10 mg/ml horseradish peroxidase. The luminescence emitted was immediately analyzed with a Beckmann-Coulter DTX800 plate reader for a period of 50 min, taking measurements for every individual well each minute. The results are shown as relative luminescence units (RLU).

For the seedling growth inhibition assays, tobacco seeds were sterilized with chlorine gas (Clough and Bent, 1998) and then germinated in Petri dishes containing 20 ml of half-strength Murashige-Skoog medium (Pronadisa, Spain) supplemented with 1% sucrose (pH 5.7). After 5 days, plantlets were transferred to 96-well plates containing 100 μl of the peptide/protein solutions in half-strength Murashige-Skoog medium. Samples were incubated in a phytotron for 7 days before, they were photographed and their fresh weight was determined.

Statistical analysis were carried out with the statistical analysis package SPSS 17.0 (IBM, Armonk, NY, United States). The applied tests are specified in figure legends for each experiment.

## Results

### A New Conserved 25 Amino Acid Peptide as an Elicitor Region of BcXyn11A

Previous results with BcXyn11A pointed to a 30-amino acid peptide as the elicitation motif, but the peptide alone, expressed in *Escherichia coli*, failed to generate any response in plants (Noda et al., 2010). Since plant elicitors are usually recognized by their most conserved domains (Felix et al., 1999), the 30-amino acid peptide was extended in order to include nearby highly conserved residues, in accordance with the sequence conservation profile of 1469 GH11 xylanases annotated in the Pfam database (Figure 1A). A new 60-residue peptide, Xyn60 (Figure 1A) was selected to be expressed in *P. pastoris*. To design Xyn60, the 30-aa peptide (Noda et al., 2010) was extended at its N-terminus by 20 residues, corresponding to the ß-strands B5 and B6 located at the palm of the catalytic cleft, and at its C terminus by 10 residues which conform the so-called “thumb” loop (Paes et al., 2012; Figure 1B). Additionally, two new peptides, 25 residues each, were designed from Xyn60 (Figure 1) and chemically synthesized. The Xyn25 peptide included the most conserved residues in all the xylanases tested, comprising also the first five residues at the N-terminus of the 30-aa fragment (Figure 1). This peptide is located in the ß-strands B5 and B6, and the connecting loop between B6 and B9 or the “cord” loop. The second peptide, Xyn25.2, comprised the remaining region of the 30-aa fragment (Figure 1) and is located in the ß-strands B8 and B9, outside of the palm (Figure 1B). The whole BcXyn11A protein was expressed in *P. pastoris* to be used as a positive control.

**FIGURE 2:**
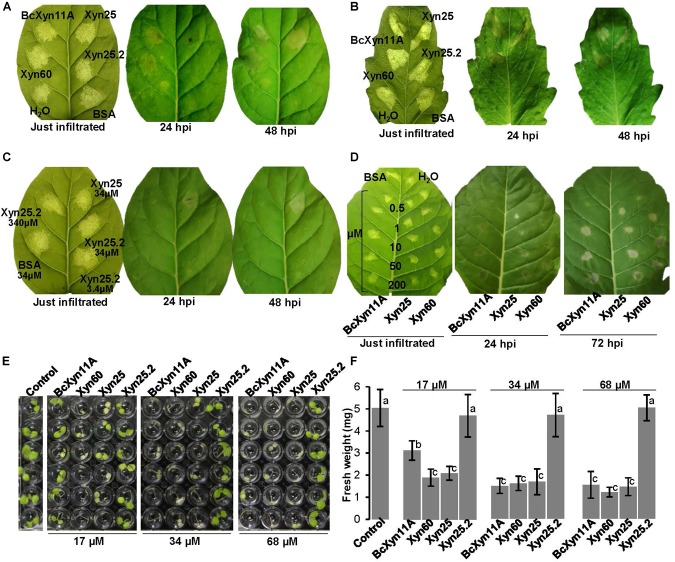
Necrosis of plant leaves and seedling growth inhibition by BcXyn11A and derived peptides. **(A,B)** Effect of the infiltration of the indicated protein/peptides on tobacco **(A)** or tomato **(B)** leaves, at indicated time after infiltration (hpi). Bovine Serum Albumin (BSA), or water, were used as negative controls. Proteins/peptides were dissolved in water at 34 μM. **(C)** Effect of the infiltration of tobacco leaves with the peptide Xyn25.2 at various concentrations, using Xyn25 and BSA as positive and negative controls, respectively. **(D)** Determination of the lowest concentration of the protein/peptides able to produce necrosis when infiltrated in tobacco leaves. The negative control BSA was infiltrated at a concentration of 200 μM. **(E)** Tobacco seedling growth inhibition assays with BcXyn11A and the derived peptides. 5-day-old seedlings were incubated for 7 days in the growth chamber with 100 μL of a solution containing half-strength Murashige-Skoog medium and the protein/peptides at the indicated concentrations. Controls contained the same medium with no protein/peptides. **(F)** Fresh weight (mean±SD, *n* = 6) of the seedlings shown in **(E)** at the end of the experiment. Different letters on bars indicate a statistically significant difference according to *t*-test (*p* = 0.05). Pictures labeled as “just infiltrated” (time zero) were taken with back illumination to show the exact region of the leaf containing the infiltrated liquid.

The necrotizing activity of BcXyn11A and the three derived peptides was first assayed at 34 μM, by infiltration in tobacco (Figure 2A) or tomato leaves (Figure 2B), using BSA at the same molar concentration or water, as negative controls. As shown previously (Noda et al., 2010), BcXyn11A was able to produce necrotic lesions in both tobacco and tomato leaves. The Xyn60 and Xyn25 peptides also produced necrotic lesions, while unexpectedly Xyn25.2 failed to do so. This peptide did not produce any tissue necrosis even if its concentration was raised up to 340 μM (Figure 2C). A dose-response experiment (Figure 2D) showed that both BcXyn11A and the two active peptides were able to produce visible necrotic lesions at a concentration as low as 1 μM, and at this concentration the two peptides showed a slightly higher necrotizing activity than the whole protein (Figure 2D).

The ability of the BcXyn11A-derived peptides to inhibit the growth of tobacco plantlets was assayed with 5-day-old seedlings (Figure 2E,F), and the results clearly showed that BcXyn11A, as well as Xyn60 and Xyn25, but not Xyn25.2, were able to cause seedling growth inhibition with clear symptoms of chlorosis. Interestingly, growth reduction was similar for BcXyn11A and the two peptides when the seedlings were treated with a 34 or 68 μM solution of the protein/peptide, while at a concentration of 17 μM, BcXyn11A seemed to be less active than the two peptides. These results strongly support that Xyn60 and Xyn25, but not Xyn25.2, contain the BcXyn11A elicitor region.

### BcXyn11A and Derived-Peptides Induce HR Symptoms

**FIGURE 3:**
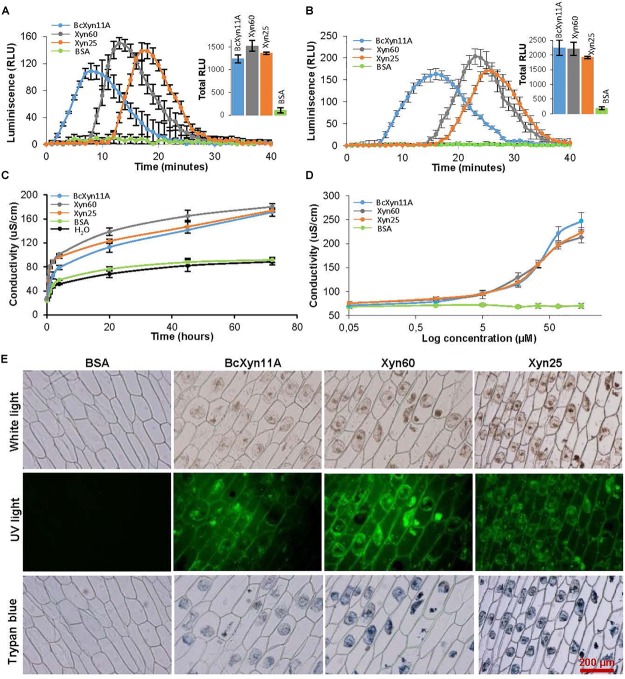
Defense-related symptoms triggered by BcXyn11A, Xyn60 and Xyn25. **(A,B)** ROS burst assays of tobacco **(A)** and tomato **(B)** leaf disks during incubation with a 17 μM solution of the indicated protein/peptides. The amount of ROS was followed by luminescence reading and is expressed as relative light units (RLU) (mean±SD, *n* = 12). Total ROS production was also calculated for each protein/peptide in both plants and is shown as an inset. **(C)** Ion leakage on tobacco leaves caused by infiltration with a 34 μM solution of the indicated protein/peptides. Conductivity (mean±SD, *n* = 3) was followed after placing disks from the infiltrated leaves in water at 45 h after infiltration. **(D)** Same as in **(C)** but various protein/peptide concentrations were used. **(E)** Onion epidermis samples were treated with a 64 μM solution of the indicated proteins/peptides for 24 h and visualized under white light (to observe cytoplasm shrinkage) and under UV-light (to observed cell autofluorescence) with a Olympus BX-50 microscope, with a U-MWIB filter for autofluorescence. The same samples were then stained with trypan blue in order to determine cell death. BSA was used at the same concentration as negative control.

To better study whether the plant defense responses induced by BcXyn11A, Xyn60, and Xyn25 were similar, several symptoms related with the HR were studied in detail. First, the typical ROS burst associated with HR (Lamb and Dixon, 1997) was assayed with tobacco and tomato leaf disks by the luminol chemiluminescence assay as explained before (Frías et al., 2016). The results (Figure 3A,B) indicated that BcXyn11A, Xyn60, and Xyn25 were able to induce a ROS burst in both plant species. Interestingly, the response was slightly faster in tobacco than in tomato, and the burst peak appeared at different times depending on the protein/peptide used in the treatment. BcXyn11A protein generated the fastest response and Xyn25 the slowest in both plants, although the total amount of ROS generated (insets in Figure 3A,B) was similar for the three peptides tested.

Ion leakage was also measured in treated tobacco leaf disks as described previously (Frías et al., 2011). In these assays, solutions of BcXyn11A, Xyn60, or Xyn25 were used to infiltrate tobacco leaves and, 4 h later, disks were excised from the infiltrated area, transferred to water, and conductivity was measured at various time points (Figure 3C). A similar increase in conductivity was detected for the leaves infiltrated with BcXyn11A, Xyn60, or Xyn25, as compared with the negative controls. In order to obtain a dose-response curve, various concentrations of the proteins/peptides were used to assay ion leakage at 45 h after infiltration, and similar results were obtained for the three molecules indicating that the concentrations producing half the maximal effect were in the range 30–40 μM (Figure 3D).

Other symptoms of the activation of plant defenses are the appearance of autofluorescence and cytoplasm shrinkage in elicitor-treated cells (Frías et al., 2011). These two effects were observed in onion epidermal cells treated with a 64-μM solution of BcXyn11A or the derived peptides (Figure 3E), while no effect was observed for the control. Besides, the treated onion cells were shown to be actually dead, by the trypan blue assay (Figure 3E).

**FIGURE 4:**
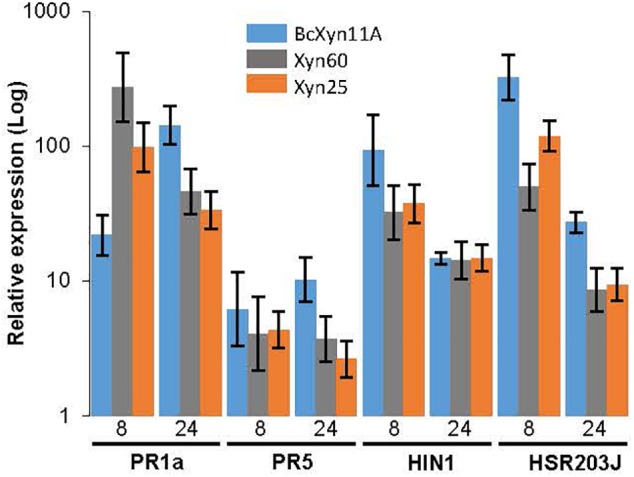
Induction of plant defense genes after infiltration with BcXyn11A, Xyn60 and Xyn25. Tobacco leaves were infiltrated with a 34 μM solution of BcXyn11A, Xyn60, or Xyn25, and the transcript levels of the indicated tobacco defense-related genes were measured by qRT-PCR at 8 and 24 h post-infiltration. Relative expression was calculated as fold increase with respect to the expression of the same genes just after infiltration with the same protein/peptides. Tobacco actin gene was used as internal reference control. Results are presented as mean±SD for three technical replicates.

The induction of four tobacco defense genes by BcXyn11A, Xyn60, or Xyn25 was also tested. For this purpose, a 34-μM solution was infiltrated in tobacco leaves and the infiltrated area was collected after 8 or 24 h to assay the expression of two HR markers (*HIN1* and *HSR203J* genes) and two genes coding for Pathogenesis Related (PR), *PR1a* and *PR5*, by qRT-PCR. The whole protein and the two peptides were able to induce the expression of these four defense-related genes as early as 8 h post-infiltration (Figure 4), although slight differences in the kinetics of induction were observed. The whole protein caused a maintained increase in *PR1a* gene expression over time, while Xyn60 and Xyn25 rapidly induced its expression to nearly 13 and 5 folds, respectively, as compared with BcXyn11A, to drop at 24 h after infiltration (Figure 4). *PR5* expression also increased over time post-infiltration with BcXyn11A, although the expression levels almost did not change after 8 h of treatment with each peptides (Figure 4). Finally, both HR markers were upregulated by the three polypeptides in a similar time course, but the expression levels of both genes induced by Xyn60 or Xyn25 were somewhat lower than that of BcXyn11A (Figure 4).

### Two Conserved Regions of Four Consecutive Amino Acid Residues Within Xyn25 Are Required to Induce the Plant Defense Response

**FIGURE 5:**
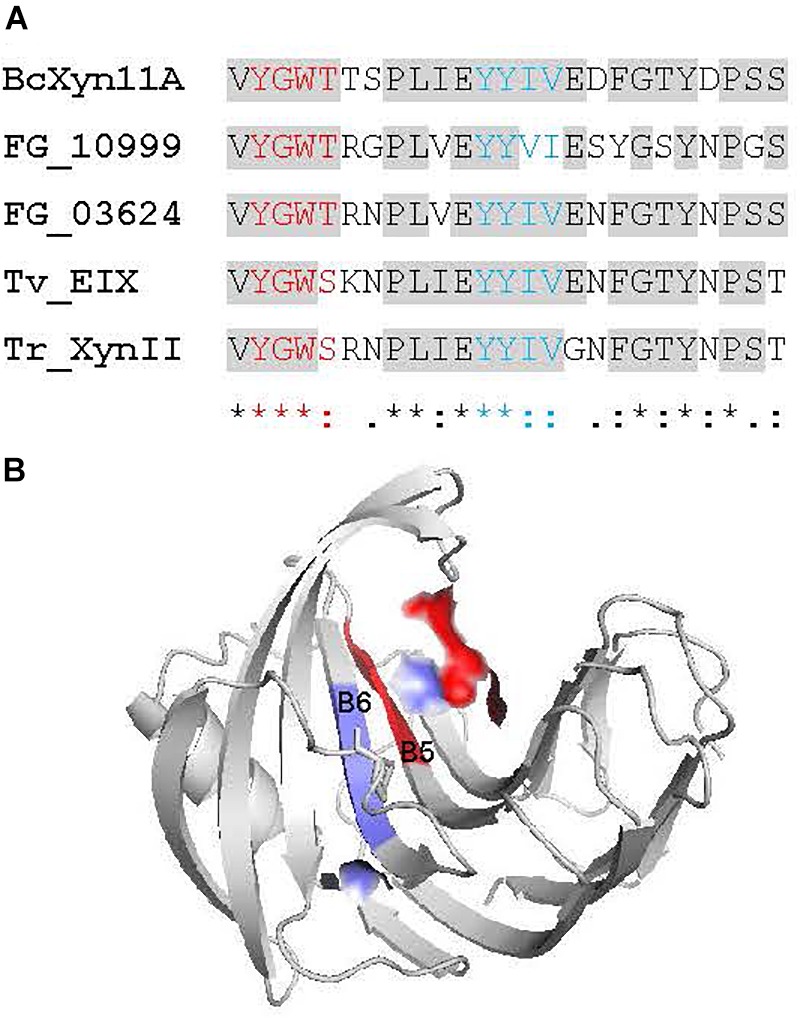
Evolutionary Conserved regions of the Xyn25 peptide. **(A)** Alignment of the Xyn25 region from five GH11 xylanases known to elicit plant defenses. BcXyn11A: *Botrytis cinerea* xylanase BcXyn11A (acc. No. Q2LMP0), FGSG_10999: *Fusarium graminearum* xylanase FGSG_10999 (Acc. No. XP_011325371), FG_03624: *Fusarium graminearum* xylanase FG_03624 (Acc. No. XP_011322077), Tv_EIX: *Trichoderma viride* xylanase EIX (Acc. No. CAB60757), Tr_XynII: *Trichoderma reesei* xylanase II (Acc. No. AAB50278). The two conserved four-amino acid regions discussed in the text are showed in red (M1) and blue (M2). **(B)** Location of the regions M1 and M2 at the surface of the BcXyn11A in the protein 3D model obtained as described in Figure 1.

**FIGURE 6:**
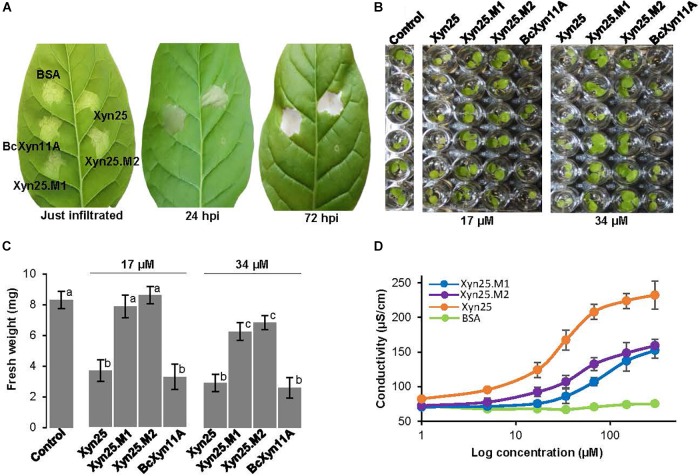
Effect of the mutation of conserved regions on the elicitor activity of Xyn25. **(A)** Effect of the infiltration of the indicated protein/peptides on tobacco leaves, at various hours after infiltration (hpi). Bovine Serum Albumin was used as negative control. Xyn25.M1 and Xyn25.M2 are mutant peptides derived from Xyn25 (see text). Proteins/peptides were dissolved in water at 34 μM. **(B)** Tobacco seedling growth inhibition assays carried out with the indicated protein/peptides as explained for Figure 2E. **(C)** Fresh weight (mean±SD, *n* = 6) of the seedlings shown in **(B)** at the end of the experiment. Different letters on bars indicate a statistically significant difference according to *t*-test (*p* = 0.05). **(D)** Ion leakage (mean±SD, *n* = 3) on tobacco leaves caused after infiltration of protein/peptide solutions at increasing concentrations, carried out as in Figure 3D.

The detailed analysis of evolutionary conservation of individual residues within Xyn25 showed two regions of four consecutive amino acids (M1 and M2) as the most conserved residues of the peptide and also of the whole BcXyn11A (Figure 1A, 5). The location of both regions at the surface of BcXyn11A showed that both are partially exposed (Figure 5B). Two new chemically synthetized peptides, Xyn25.M1 and Xyn25.M2 (Supplementary Figure S1), were generated by substituting each amino acid of regions M1 or M2 by alanine residues. Neither peptide was able to produce visible lesions when infiltrated in tobacco leaves even after 72 h (Figure 6A), while Xyn25 and the whole BcXyn11A did so at the same molar concentration (34 μM). Tobacco seedlings growth was slightly inhibited by the two new peptides, but only when high concentrations were used (Figure 6B,C). A dose-response curve was obtained infiltrating different concentrations of each peptide in tobacco leaves and measuring the ion leakage (Figure 5D). Both peptides showed partial activity compared with Xyn25 at all concentrations tested. The concentrations of Xyn25.M1 and Xyn25.M2 that resulted in 50% of maximum electrolyte leakage were similar to those determined for BcXyn11A and Xyn25 (30–40 μM), while the maximum effect caused by the two mutant peptides was about 65–68% that of Xyn25. These results suggest that the two well-conserved four-amino acid regions in Xyn25 contribute to the induction of plant defenses, and therefore are required for the full elicitor activity of Xyn25.

## Discussion

BcXyn11A had been characterized as a pathogenic factor of *B. cinerea*, being able to hydrolyze xylan and, irrespective of its enzymatic activity, induce necrosis of the plant tissue (Brito et al., 2006; Noda et al., 2010). In this work, the plant response to BcXyn11A has been deeply studied. The protein is involved in the transcriptional activation of plant defense-associated genes within 8 h after infiltration in tobacco leaves (Figure 4): PR proteins, salicylic acid-responsive PR1a and PR5, were upregulated over time, but HIN1 and HSR203J proteins, associated in HR cell death, decreased their expression after 24 h of infiltration. As expected, the overexpression of these genes had a fitness cost in plants causing a 30–70% decrease in the fresh weight of tobacco seedlings treated with the protein, in a concentration-dependent manner (Figure 2E,F). Moreover, this defense response produced a rapid accumulation of ROS (Figure 3A) triggering cell death as shown by the rapid electrolyte leakage detected on infiltrated tobacco leaves (Figure 3C,D), as well as the autofluorescence, cytoplasm shrinkage, and trypan blue-stained cells observed in onion epidermis incubated with BcXyn11A (Figure 3E). All these effects are hallmarks that define the HR (Mur et al., 2008).

Besides BcXyn11A, other GH11 xylanases have been reported to elicit plant defenses. These include *T. viride* EIX (Dean et al., 1989), *T. reesei* xylanase II (Enkerli et al., 1999), and more recently, *F. graminearum* xylanases FG_03624 and FGSG_10999 (Tundo et al., 2015). In searching for the region or regions acting as PAMP in these proteins, at least two different elicitor motifs have already been proposed, the pentapeptide TKLGE, located near the catalytic cleft in xylanase II and EIX (Rotblat et al., 2002), and a 30-residue peptide comprising β-strands B8 and B9 and the “cord” loop (Figure 1) of BcXyn11A (Noda et al., 2010). In both cases, indirect evidences pointed to the implication of these regions in the induction of plant defenses, although also in both cases, the isolated 5- or 30-residue peptides did not produce any effect on the plants tested. Therefore, in this work, three small peptides (Xyn60, Xyn25, and Xyn25.2) were designed to include highly conserved regions nearby the 30-amino acid fragment of BcXyn11A (Figure 1A). Initial characterization of the three of them showed that Xyn60 was able to induce necrosis in infiltrated tobacco and tomato leaves and to inhibit the growth of tobacco seedlings, resembling the results obtained with BcXyn11A (Figure 2). However, only Xyn25 produced results similar to Xyn60 (Figure 2), discarding the region of BcXyn11A included in Xyn25.2 as a putative elicitor region. Further characterization of Xyn60 and Xyn25 showed that, in most of the tests performed, both peptides displayed almost the same HR-eliciting activity as BcXyn11A (Figure 3, 4). The most pronounced difference was seen in the time lag of ROS production for Xyn60 and Xyn25 in infiltrated tomato and tobacco leaves (Figure 3A,B). These slight differences could be due to their intrinsic differences in size and charge, and also to the final conformation of each molecule, which may influence the ease or speed with which they are recognized. Hence, the well-conserved 25-residue region included in Xyn25 and located close to the enzyme’s active site (Figure 1) appears to be mostly responsible for the elicitor activity of BcXyn11A. Although the minimum elicitor motif has not been identified in this work, it was shown that mutations in conserved residues along Xyn25 affect elicitor activity (Figure 6), thus indicating that a peptide much smaller that Xyn25 that retains full elicitor activity might not be possible. On the other hand, the use of bigger peptides will unlikely result in a significant increase in elicitor activity, as the effects of Xyn25, Xyn60, and BcXyn11A were so similar. The alignment of the Xyn25 sequence for the five xylanase elicitors described so far (Figure 5) shows a high degree of conservation in this region, with 48% identity and 98% similarity. This result fits well with the presumptive evolutionary advantage conferred by a single perception system able to detect the same protein pattern in multiple pathogens. Besides, a BLAST search using the Xyn25 sequence as query against the non-redundant protein sequences database at NCBI, resulted in 384 hits with 80% or more amino acid identity with the query, in a region of at least 23 residues (searched on December 26, 2018). Most of these hits are annotated as GH11 xylanases.

A detailed analysis of the sequence of Xyn25 revealed two highly conserved small regions spaced six residues apart, among GH11 xylanases (Figure 1). The four amino acids of each region were changed to alanine residues generating two new peptides (Xyn25.M1 and Xyn25.M2) that were unable to induce necrosis in tobacco leaves, although had a slight but significant effect on the growth of tobacco seedlings at high concentrations (Figure 6A,B). Besides, each new peptide affected the cell membrane integrity detected by increased electrolyte leakage, though also to a lesser extent than Xyn25 (Figure 6C). Therefore, Xyn25.M1 and Xyn25.M2 retained only partly the activity of Xyn25 at all concentrations tested, thus revealing that the two well-conserved four-amino acid regions in Xyn25 contribute to the induction of plant defenses and are required for its full elicitor activity.

Site directed mutagenesis of the two Glu residues essential for catalysis has shown that the elicitation activities of BcXyn11A, EIX, and xylanase II are independent of their xylan-hydrolyzing activity (Enkerli et al., 1999; Furman-Matarasso et al., 1999; Noda et al., 2010), as would be the case if xylan oligomers were the actual elicitors. Indeed, mutant proteins were identified in these three studies that had completely lost the enzymatic activity, but retained the elicitation activity. On the other hand, certain particular mutations in these two Glu residues in EIX and xylanase II (Enkerli et al., 1999; Furman-Matarasso et al., 1999; Noda et al., 2010) were also able to diminish the eliciting activity, sometimes to almost zero. E86 in EIX is located within the region corresponding to Xyn25 in EIX, in consonance with the role of Xyn25 as elicitation motif proposed here, but E177 in EIX is far from it in the amino acid sequence although it is also located in the active site. Anyway, mutation of the residues corresponding to EIX E86 and E177 in *B. cinerea* BcXyn11A, to either Ser or Gln (Noda et al., 2010), did not affect the elicitor activity in a significant way.

This is the first case, therefore, in which a xylanase peptide has been undoubtedly shown to display the capacity for inducing the plant defenses by itself. As expected, Xyn25 includes the region of BcXyn11A that shows the highest degree of evolutionary conservation, and the substitution of the most-conserved residues within Xyn25 by alanine residues greatly diminishes its eliciting activity. Although at this point the existence of additional elicitor regions in BcXyn11A, beside Xyn25, cannot be ruled out, the similarity of responses observed for the whole protein and the two peptides do indicate that Xyn25 is able to reproduce the elicitation activity of xylanase by itself. Sequence similarity between BcXyn11A and EIX points to LeEix1/2 as having a role in BcXyn11A/Xyn25 perception, although the existence of additional/alterative receptors cannot be ruled out and may perhaps explain the differences observed for the timing of the ROS burst (Figure 3). Additionally, as BcXyn11A contributes to *B. cinerea* virulence mostly with its elicitor activity (Noda et al., 2010), it follows that Xyn25 should also be able to contribute to virulence when expressed in the fungus, a hypothesis that may be interesting to test in the future.

## Author Contributions

MF and MG drafted the initial manuscript. All authors participated in the design of the experiments as well as the analysis/evaluation of the results, and edited and approved the final version of the manuscript.

## Conflict of Interest Statement

The authors declare that the research was conducted in the absence of any commercial or financial relationships that could be construed as a potential conflict of interest.

## Footnotes

1 http://www.rcsb.org/pdb

2 https://swissmodel.expasy.org

3 http://fungi.ensembl.org/Botrytis_cinerea

4 http://www.cbs.dtu.dk/services/
