# Supplementary material for: A 25-Residue Peptide From Botrytis cinerea Xylanase BcXyn11A Elicits Plant Defenses
Source: Front Plant Sci. 2019 Apr 16;10:474. doi: 10.3389/fpls.2019.00474 (PMC6477079; doi:10.3389/fpls.2019.00474)
Supplement: Supplementary file 1 [file Data_Sheet_1.PDF]

Supplementary Material

A 25-residue peptide from *Botrytis cinerea* xylanase BcXyn11A elicits plant defenses

Marcos Frías, Mario González, Celedonio González and Nélida Brito*

***Correspondence:**
Nélida Brito
Email: nbrito@ull.edu.es

Supplementary Table 1.

Table S1: List of primers used in this work

Primer ID	Sequence (5' – 3')	Details
Xyn11A-EcoRI-FW	CGGAATTCCCCGTCAGCGAGAACTTG	Amplification of *Bcxyn11A* gene sequence for expression in *Pichia pastoris*
Xyn11A-XbaI-RV	GCTCTAGACCAGAAACAGTGATGGAAGCG	
Xyn60aa-EcoRI-FW	CGGAATTCGTCTATGGTTGGACTACCTCC	Amplification of the DNA sequence coding Xyn60 for expression in *P. pastoris*
Xyn60aa-XbaI-RV	GCTCTAGACCCTTGAAGGTAGCAGTTCCTTG	
AOX 5'	GACTGGTTCCAATTGACAAGC	Check *P. pastoris* strains
NtActin-FW	GAACTCGAGACGTCAAAGACC	Amplification of approximately 100 bp of the indicated *N. tabacum* genes for qRT-PCR experiments
NtActin-RV	AAAAGGACCTCAGGACAACG	
NtHIN1-FW	CTGCAACCCATGTAGCTGTC	
NtHIN1-RV	GGTCGAAGAACGAGCCATAG	
NtHSR203-FW	TGCCGTCAAAGATGTAGTCG	
NtHSR203-RV	CAGCATGGCTGACACAAAAG	
NtPR1A-FW	ATGCGCAAAATTATGCTTCC	
NtPR1A-RV	TCATCGACCCACATCTCAAC	
NtPR5-FW	CTCATGCTGCCACTTTTGAC	
NtPR5-RV	CTCCAAGATTGGCCTGAGTC	

Supplementary Figure 1.

A

BcXyn11A (Expressed in *Pichia pastoris*; 226 aa, 24.33 kDa)

PVSENLNVLQERALTSSATGTSGGYYYSFWTDGSGGVTYS
NGANGQYAVSWTGNKGNFVGGKGWAVGSERSISYTGSYK
PNGNSYLSVYGWTTSPLIEYYIVEDFGTYDPSSAATEIGSVT
SDGSTYKILETTRTNQPSVQGTATFKQYWSVRTSKRTSGT
VTTANHFAAWKKLGLTLGSTYNYQIVAVEGYQSSGSASITV
S**GLEQKLISEEDLNSAVDHHHHHH**

Xyn60 (Expressed in *P. pastoris*; 83 aa, 9.2 kDa)

VYGWTTSPLIEYYIVEDFGTYDPSSAATEIGSVTSDGSTYKIL
ETTRTNQPSVQGTATFK**GLEQKLISEEDLNSAVDHHHHHH**

Xyn25 (Chemical synthesis; 25 aa, 2.9 kDa)
VYGWTTSPLIEYYIVEDFGTYDPSS

Xyn25.2 (Chemical synthesis; 25 aa, 2.6 kDa)
AATEIGSVTSDGSTYKILETTRTNQ

Xyn25.M1 (Chemical synthesis; 25 aa, 2.8 kDa)
VAAAATSPLIEYYIVEDFGTYDPSS

Xyn25.M2 (Chemical synthesis; 25 aa, 2.6 kDa)
VYGWTTSPLIAAAAVEDFGTYDPSS

B

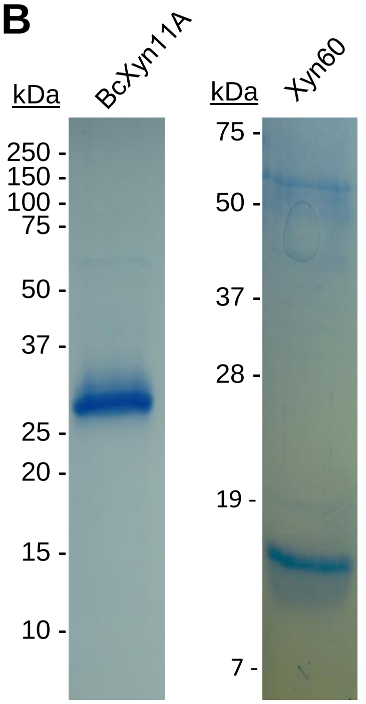

Fig. S1. Proteins and peptides used in this work. A) Amino acid sequences of BcXyn11A, Xyn60 (as expressed in *Pichia pastoris*), Xyn25, Xyn25.2, Xyn25.M1 and Xyn25.M2, the number of amino acid residues and the expected molecular weight. The c-*myc* and 6xHIS sequences are shown in red when present, putative *O*-glycosylated residues were predicted according to NetOglyc 4.0 server (www.cbs.dtu.dk) and are shown in blue. Modified regions of Xyn25.M1 and Xyn25.M2 peptides are underlined. B) SDS-PAGE (stained with Coomassie Blue G250) of purified BcXyn11A and Xyn60. The slight increase in the observed molecular weight, as compared with that expected from the amino acid sequence, could be due to *O*-glycosylation introduced by *P. pastoris*.
